# Effects of Different Oral Doses of Sodium Chloride on the Basal Acid-Base and Mineral Status of Exercising Horses Fed Low Amounts of Hay

**DOI:** 10.1371/journal.pone.0168325

**Published:** 2017-01-03

**Authors:** Annette Zeyner, Kristin Romanowski, Andreas Vernunft, Patricia Harris, Ann-Marie Müller, Carola Wolf, Ellen Kienzle

**Affiliations:** 1 Institute of Agricultural and Nutritional Sciences, Group Animal Nutrition, Martin-Luther-University Halle-Wittenberg, Halle (Saale), Germany; 2 Chair for Nutritional Physiology and Animal Nutrition, University of Rostock, Rostock, Germany; 3 Leibniz Institute for Farm Animal Biology, Dummerstorf, Germany; 4 Equine Studies Group, Waltham Centre for Pet Nutrition, Waltham-on-the-Worlds, United Kingdom; 5 State Office for Agriculture, Food Safety and Fishery Mecklenburg-Western Pomerania, Rostock, Germany; 6 Institute of Physiology and Animal Nutrition, Ludwig-Maximilian University Munich, Munich, Germany; Qom University, ISLAMIC REPUBLIC OF IRAN

## Abstract

The provision of NaCl, according to current recommendations, to horses in moderate work has been shown to induce immediate postprandial acidosis. The present study aimed to clarify whether this NaCl induced acidosis i) persists beyond the immediate postprandial period, and ii) is still present after a 2 week adaptation period. Six adult warmblood mares in moderate work received daily 1.00 kg hay per 100 kg body weight (bwt) only together with 0.64 kg unprocessed cereal grains/100 kg bwt.d as fed basis. Using a 3x3 Latin Square, either 0 (NaCl-0), 50 (NaCl-50) or 100 (NaCl-100) g NaCl/d were fed together with the concentrates in two equal doses for 3 weeks. During the final week, a mineral digestibility trial was undertaken. The middle sodium and chloride intake (NaCl-50) at least met the most common recommendations for moderate work. Morning (7:00 AM) urine and venous blood samples were collected on days 0, 1–4, 8, and 15, and analysed for pH, acid-base status, creatinine and electrolyte concentrations. Fractional electrolyte clearances (FC) were determined. Mean apparent sodium digestibility ranged between 60–62% whereas chloride digestibility was consistently above 94%. Supplementing 100 g but not 50 g of NaCl resulted in significant reduction of blood pH and base excess as well as urinary pH and urine acid excretion. Both 50 g and 100 g NaCl supplementation caused a significant reduction in base and net acid-base excretion, urine density and potassium concentration, but increased urine sodium concentration and the FC of sodium and chloride (*P* < 0.05). This suggests that a high proportion of the recommended salt doses is excreted renally. The above effects of NaCl supplementation persisted over the 2 week measurement period. Results suggest that feeding 100 g NaCl to moderately exercising horses results in mild metabolic acidosis, whereas feeding 50 g according to current recommendations resulted in compensated acidosis.

## Introduction

The level of sodium and chloride in typical horse forages and feeds (especially non-commercially manufactured) is low [1]. The recommended intakes of sodium and chloride for exercise performance [2], [1] are therefore unlikely to be met by non-supplemented diets and NaCl supplementation is commonly recommended.

Since equine sweat is rich in sodium, potassium and chloride [3], [4], [5], [6], [7], [8], diets marginal in these electrolytes may cause disturbances of fluid and mineral homeostasis and therefore potentially health problems in exercised horses [9]. However, recently the value of supplementing, especially in the non-endurance horse, at the currently recommended levels has been questioned and this topic has become highly controversial [10]. Lindner [11] reported a reduction of the extracellular volume in horses receiving an artificially sodium depleted ration. Furthermore, plasma aldosterone concentration was reduced in horses in training when consuming diets with low amounts of sodium [12]. On the other hand, eventing horses receiving no or ‘inadequate’ amounts of additional NaCl supplementation showed persistent good performance and apparently undisturbed health [13]. It has been reported that the voluntary intake of NaCl ([12]: 0–62 mg/kg bwt.d) is frequently lower than required to meet the currently recommended intakes for horses in work [12] although it might be sufficient for those in little or no work [14]. Back in 1890, Smith [15] reported that horses working without any additional salt intake retained apparent excellent health. He stated that ‘no matter how necessary sodium may be for herbivore in the wild state (as travellers would have us believed) it is perfectly certain that under domestication they can be maintained in perfect health without receiving any more than is normally contained in their food.’.

Interestingly, Smith [15] also demonstrated that working horses adaptively reduced the renal excretion of both sodium and potassium, relative to maintenance conditions. A similar effect was later reported by Coenen [7] who found that whilst the electrolyte concentrations in the sweat did not change significantly even when in a state of sodium, potassium or chloride depletion, renal electrolyte output was lowered (by 13–39% for chloride) in such instances for the three days following exercise. In addition, sweat sodium concentration does not appear to be entirely constant. For instance, sweat sodium concentration was shown to increase with increasing sweating rate [16], but to decrease after a period of three weeks of training under hot and humid conditions [17]. Breed or even individual differences may also play a role. Spooner et al. [18] observed a comparatively low sodium content in the sweat of Arabian horses. Evaporation of water during sweat collection may lead to an overestimate of the electrolyte content of sweat [5]. In addition, sweat losses show considerable variation, and they are not very closely linked to the intensity of work but also to other factors such as environmental temperature and individual horse [19].

The obvious contradiction between currently recommended requirements and observed free choice intake of horses remaining apparently perfectly healthy may arise for a number of reasons including the following numbered 1–7. 1) Current recommendations overestimate the actual requirements (for maintenance requirement see: [20]; for discussion of sweat losses see [19]). 2) Consuming salt causes some adverse effect leading to an acquired taste aversion as shown in sheep [21] and rats [22]. 3) Horses can to some extent regulate their electrolyte losses through internal strategies such as modifying renal [15], [7], [23] and faecal [12], [23] losses of electrolytes (with different proportions of renal to faecal flexibility for the respective elements) and do this in preference to modifying their electrolyte intake. 4) Horses have the ability to exchange ions with body stores like postulated for humans [24]. 5) Horses cannot replace the salt losses by sweat after strenuous exercise within a few hours. 6) Horses have no innate sense or appetite as to their sodium or chloride requirements (what is rather unlikely). 7) Finally there are sub-clinical consequences to not feeding according to recommendations. It is important to note, that providing NaCl is not without risk, for example in other species high salt intake may modify insulin sensitivity [25] and may increase the risk of developing gastric ulcers as well cancer [26]. Holbrook et al. [27] investigated the impact of repeated oral hypertonic electrolyte administration in horses at rest (mimicking the practices that are common during endurance competitions) and found a significant increase of both, ulcer number and severity scores in the non-glandular stomach. Whilst this work needs to be repeated under different conditions it does suggest that this could be a potential consequence of inappropriate salt administration.

One of the other reasons, however, why the oral supplementation of NaCl is controversial, is that it may cause a metabolic acidosis [28], [29], [30] and adversely affect the renal excretion of sodium, chloride and possibly other minerals [28], [29], [30]. Such an effect has been shown in humans in whom the stepwise increase of NaCl intake led to a low-grade metabolic acidosis with increased renal output of calcium and exaggerated bone resorption [31]. In horses, an acidifying diet also elevated urinary excretion of calcium [32].

Metabolic acidosis induced by an oral NaCl load has only been evaluated in the horse over a relatively short period of supplementation (4 days) and in the immediate postprandial period [28], [30]. We hypothesize, that NaCl given at the currently recommended levels to moderately exercised horses would cause a persistent reduction of urine and possibly blood pH. This hypothesis was tested with 2 doses of NaCl from which the lower dose (50 g NaCl/d) corresponds to current recommendations for compensation of electrolyte losses for the respective work [2], [1] and the higher one (100 g NaCl/d) is more close to established practice. A further aim of the study is to see to what extent the fractional urinary clearance of sodium and chloride would be affected by such levels of supplementation.

## Materials and Methods

The study was approved by the animal welfare committee of Mecklenburg-Western Pomerania (LALLFM-V/TSD/7221.3.2.1–0208).

### Animals

Six fit warmblood type mares with a body weight of 627 ± 33.5 kg and an age of 12.8 ± 4.8 years undertook the study. Throughout the study, the horses were exercised according to a protocol for moderate work as formerly performed ([33]; per horse and day for 5 days a week, except on test days, approximately 15 min walk, 25 min trot and 5 min canter). The mares had been exercised at this intensity for at least one year prior to this study.

The horses were kept individually in boxes with wooden shavings as bedding. During the adaptation period (d13—d16) and the sampling period (d17 –d21) of the digestibility trials they were housed on rubber mats.

### Diets

The horses were fed a core diet consisting of meadow hay, cereal grains (unprocessed oats and barley in a 3:2-ratio) and a NaCl-free mineral mix. The diet had been calculated to provide 1.4-fold the maintenance level of metabolizable energy (metabolizable energy in the feed according to Kienzle and Zeyner [34] and GfE [1]; energy requirement for maintenance according to Kienzle et al. [35] and GfE [1]: 0.52 MJ ME/kg dry matter, DM) which corresponds to the requirements for medium work according to NRC [2]. The feedstuffs were given in two equal per day (07:30 AM and 4:30 PM) consisting of 24.75 g/kg bwt^0.75^ (5.0 g/kg bwt) meadow hay and 15.80 g/kg bwt^0.75^ (3.2 g/kg bwt) of the mix of oats and barley grains each as fed. The amount of meadow hay was limited because several studies showed that hay and other green plant material modifies acid-base-balance in horses [36], [32], [37]. The concentrates were given just before the hay. The NaCl-free mineral mix (100 g) was provided daily with the morning concentrates. The mares had free access to tap water *via* automatic water suppliers to which they were fully adapted. The analyzed chemical composition of the feedstuffs including the tap water is given in Table 1. The core diet provided the following amounts of the major elements (in mg/kg bwt.day): Ca 66, P 61, Mg 29, Na 13, K 176, Cl 57, S 34 (S as tabulated; [38]). The dietary cation-anion-difference (DCAD) was calculated from the equation suggested by Kienzle et al. [36]: DCAD (in mmol/kg DM) = 49.9 Ca + 82.3 Mg + 43.5 Na + 25.6 K– 59.0 P– 28.2 Cl– 62.4 S (minerals in g/kg DM). The DCAD was 180, 175 and 169 mmol/kg of DM for NaCl-0, NaCl-50, and NaCl-100, respectively.

**Table 1:** Analyzed content of proximate nutrients and major elements in the experimental feedstuffs and mineral content of the tap water.

Item		Cereal grains	Meadow hay	Mineral-mix^1^	Tap water
Dry matter	[%, as fed]	94.6	94.3	89.4	
Crude ash	[% DM]	2.6	4.8	7.5	
Crude protein	[% DM]	15.1	12.8	10.1	
Acid ether extract	[% DM]	4.7	1.4	4.9	
Crude fiber	[% DM]	7.6	32.9	2.0	
Metabolizable energy^2^	[MJ/kg DM]	13.2	7.0	0.0	
Calcium	[g/kg DM]	1.14	5.43	17.41	101.0^3^
Phosphorus	[g/kg DM]	4.83	3.75	2.80	
Sodium	[g/kg DM]	0.19	1.54	0.81	13.4^3^
Potassium	[g/kg DM]	6.70	13.73	4.32	1.9^3^
Magnesium	[g/kg DM]	1.25	2.14	3.29	10.5^3^
Chloride	[g/kg DM]	0.83	6.75	1.46	29.9^3^
Sulfur	[g/kg DM]	2.14^4^	2.00^4^	2.58^5^	

^1^ nutrient content according to the manufacturer (per kg): Lysine, 2 g; Vitamin A, 400,000 international units (IU); Vitamin D_3_, 40,000 IU; Vitamin E, 5 g; Vitamin K_3_, 75 mg; Vitamin B_1_, 240 mg; Vitamin B_2_, 200 mg; Vitamin B_6_, 100 mg; Vitamin B_12_, 500 mcg; nicotinic acid amide, 300 mg; Ca-pantothenate, 200 mg; folic acid, 50 mg; biotin, 5,500 mcg, choline chloride, 1,400 mg; ferric sulphate 2,000 mg; copper as sulphate, 700 mg; zinc, 3,000 mg; zinc as oxide, 2,700 mg; zinc as acetate, 300 mg; manganese as oxide, 1,600 mg; iodine as iodate, 15 mg; selenium as selenite, 25 mg; cobalt as carbonate 12 mg (product name and manufacturer: SALVANA Pferdemineral—special blend for study purposes, SALVANA Tiernahrung GmbH, Elmshorn, Germany).

^2^ calculated according to Kienzle and Zeyner (2010).

^3^ mineral content of the tap water in mg/l.

^4^as tabulated (Nehring et al. 1972).

^5^as analysed.

### Study design

After 4 weeks of adaptation to the core diet, the horses were randomly paired and fed according to a 3 x 3 Latin square design (3 diets x 3 pairs of mares over in total 3 study periods). The three trial diets were the core diet alone (NaCl-0) and the core diet supplemented with NaCl at either 50 (NaCl-50), or 100 (NaCl-100) g per day. NaCl was given in loose form in two equal meals together with the cereal grain concentrate.

The study comprised 3 trial periods. Each period lasted 28 days with 21 days of feeding the respective trial diet (d1 –d21: NaCl-0, NaCl-50 or NaCl-100) followed by 7 days of NaCl-free feeding (washout period: d22 –d28: NaCl-0).

### Sampling

The horses were weighed (AWE460, PAARI Wagen Erfurt, Germany) at the beginning and the end of the study prior to the morning meal. To determine digestibility, the indicator method with 4N HCl-insoluble ash [39], [40], [41] as described in detail by Fuchs et al. [42] was used. For this, morning faeces were sampled over a period of 5 days (d17—d21). The faeces were taken directly from the *ampulla recti*, thoroughly mixed and a sub-sample of 300 g per horse per day was frozen (- 20°C) for later analysis.

For each period, blood and urine samples were collected when the core diet only was fed (d0), during the first 4 days (d1, d2, d3, d4) of feeding the study diets (NaCl-0, NaCl-50 or NaCl-100) as well as after 1 and 2 weeks (d8, d15) of feeding the respective diets. In every case this was done prior to the morning meal which was at 7:30 AM. Blood was sampled by puncture of the *vena jugularis externa* initially for blood gas analysis by use of a heparinised syringe (Monovette^®^, Sarstedt AG&Co., Nürnbrecht, Germany). The rectal temperature of the individual horse was measured simultaneously. Blood for serum (Monovette^®^, Sarstedt AG&Co., Nürnbrecht, Germany) was then collected. The samples were centrifuged for 8 min at 3,000 x g. Urine samples were collected after a few minutes of lunging in walk and slow trot *via* an aseptic bladder catheter (Eruplast^®^, Rüsch AG, Kernen, Germany). Plasma, serum and urine were immediately frozen (- 20°C) for later analysis.

### Measurements, chemical analysis and calculations

#### Environmental conditions

Ambient temperature (°C) and relative humidity (%) in the stable were recorded every 30 minutes by the automatic system testo 175-H2 (Testo AG, Lenzkirch, Germany).

#### Water intake

The water intake of each individual horse was automatically detected by a water flow meter which was installed at every drinking trough. The water consumption was recorded daily before the morning meal and expressed as water intake over 24 hours.

#### Feed and faeces

The content of dry matter and proximate nutrients in feed and faeces samples were analysed according to VDLUFA [43], 4N HCl-insoluble ash was determined as described by Fuchs et al. [42]. Major elements in feedstuffs and faeces were detected using the following methods as previously described by Stürmer [29] and Kienzle et al. [36]: Na, K, Ca by flame photometry (EFOX 5053, Eppendorf AG, Hamburg, Germany); P by spectrophotometry (GENESYS 10 UV, Thermo Specitronic Rochester, USA); Cl *via* ion-sensitive electrode (Eppendorf Chloridmeter, Eppendorf AG, Hamburg, Germany); Mg by atomic absorption spectrometry (Perkin Elmer, Waltham, UK).

#### Blood

The pH value, carbon dioxide tension (pCO_2_), standard base excess (SBE), standard bicarbonate (SBC) and ionized Ca (Ca^++^) were determined by calibrated and adjusted (according to the rectal temperature of the individual horse) blood gas analyzer (RAPIDLAP 348, Bayer Diagnostics, Munich, Germany). For adjustment, a standard temperature of 37°C was pre-set. Blood serum was analysed by use of the automated analyser Cobas Mira Plus (Roche Diagnostics, Grenzach-Wyhlen, Germany). Specifically this was done photometrically for inorganic phosphorus (P_i_, with ammonium molybdate), Mg by xylidylblue, creatinine with a kinetic assay according to Jaffé, total protein (TP) by the biuret method, lactate (La^-^) with lactate oxidase, Na^+^, K^+^, and Cl^-^ with ion-sensitive electrode. Ca was analysed by flame photometry with Flapho 4 (Carl Zeiss, Jena, Germany).

#### Urine

The urine density was measured by a density hydrometer (Assistent Laborpartner). The pH value was detected potentiometrically using an automated pH analyser (pH-Meter HI8314, Hanna Instruments, Vöhringen, Germany). The fractionated net acid base excretion (NABE), which describes the reciprocal of the total renal H^+^ excretion (NABE in mmol/L urine), was determined by titrating a standardized amount of urine with either HCl or NaOH to pH 3.5 or 7.4, respectively. From this the concentration of bases (HCl consumption) and of acids (NaOH consumption) can be calculated. In addition, ammonium ions are determined by modified titration with NaOH. Finally, NABE was calculated as follows: NABE = bases–acids–ammonium ions in mmol/l [44], [33]. The estimated daily urine volume (dUV) was calculated from the urinary creatinine content as described by Meyer and Stadermann [45]: dUV (ml/kg bwt.d) = 0.24 (24.30 + 1243.77 / urine creatinine concentration (mmol/L). The base acid quotient (BAQ) was obtained by division of the concentration of bases by that of total acids, including ammonium ions in the urine. Calcium, phosphorus, magnesium, sodium, potassium, chloride and creatinine in the urine samples were analysed as described above for blood serum by the automated analyser Cobas Mira Plus (Roche Diagnostics, Grenzach-Wyhlen, Germany) with the following coefficients of variability for the duplicated measurements: Ca^++^ 3.57%, P_i_ 5.44%, Mg^++^ 5.23%, Na^+^ 2.04%, K^+^ 2.24%, Cl^-^ 2.19%, creatinine 4.68%, TP 3.40%, La^-^ 5.29%. To calculate the FC of calcium, inorganic phosphorus, magnesium, sodium, potassium and chloride, creatinine and the individual electrolyte concentrations in blood serum and urine were used [46].

### Statistics

Statistical examination was done using the software package SPSS (Version 19.0, IBM SPSS). The horses bwt’s at the beginning and the end of the study were compared by t-test for paired samples. Body temperature, respiratory rate as well as blood and urine variables were checked for normal distribution (Kolmogorov-Smirnov test) and then subjected to analysis of variance to determine the impact of the dietary treatment (‘variant’; NaCl-0, NaCl-50, NaCl-100), the time within the trial period with repeated measurements (‘time’; d0, d1, d2, d3, d4, d8, d15) and the interaction of both. Prior to this, results were related to an uniform starting level (d0 = ‘1’). Means ± pooled s.d. are given as back transformed results. For statistical analysis of digestibility coefficients, ‘variant’ was the only factor of variance. *Post hoc* comparison of means was performed using the SNK test. The level of statistical significance was pre-set at *P* < 0.05.

## Results

### Environmental conditions

The measured environmental conditions throughout the study period (in the stable; averaged over 24 hrs/d) were as follows: relative air humidity, 76.2 ± 6.00% (range from 59 to 86%); ambient temperature, 9.4 ± 2.5°C (range 2.5 to 14.1°C).

### General observations and body weight

All horses remained clinically healthy with no obvious change in behaviour throughout the entire study. Body temperature and respiratory rate (9.3 ± 0.43 min^-1^) were within the normal range and there was no effect of dietary treatment. There was also no change in bwt (*P* > 0.05) being 628 ± 28.9 kg at the end of the study compared to 627 ± 33.5 kg at the beginning.

### Feed intake

The hay and cereal grain concentrates were always eaten completely in the control group. Concentrates enriched with NaCl were also consumed completely but the horses seemed to take longer to eat them (data not recorded).

### Water balance

The mean water intake (all diets, trial days and horses) over 24 hrs was 30.7 ± 9.55 litres with no significant effect (*P* > 0.05) of salt addition. On an individual horse basis three horses showed no increase in water intake at either level of salt addition, one horse only increased water intake in response to the high dose and the other 2 increased to a similar level with both doses (mean of all horses within the respective diet, in L/d: NaCl-0, 28.2 ± 4.90; NaCl-50, 31.0 ± 7.65; NaCl-100, 30.9 ± 5.39). Mean faecal dry matter was unaffected by salt. It ranged between 22 and 23%. The same was true for faecal sodium excretion. The diet, however, influenced urine density (*P* = 0.001) and calculated urine volume (*P* = 0.043). The urine density was lower when either dose of NaCl were supplied compared to NaCl-0 (*P* < 0.05; Table 2).

**Table 2:** Biochemical variables in the urine of six horses within the individual feeding variants and days of dietary treatment, respectively (mean ± pooled SD; *P* values from analysis of variance).

	*P* value			Mean										Pooled SD
	for variance-factor			diet [g NaCl/d]			time [day of treatment]							
	diet(d)	time(t)	d*t	0	50	100	0	2	3	4	5	8	15	
pH	0.036	0.262	0.969	7.42^a^	7.46^a^	7.12^b^	7.56	7.42	7.18	7.23	7.40	7.46	7.07	± 0.653
Density	0.001	0.575	0.991	1.034^a^	1.030^b^	1.027^b^	1.031	1.032	1.032	1.029	1.024	1.029	1.030	± 0.000
Ca	0.292	< 0.001	0.278	22.0	27.0	21.0	22.5^b^	22.5^b^	30.2^a^^b^	12.9^b^	13.0^b^	19.4^b^	43.1^a^	± 20.77
Na	<0.001	0.666	0.948	36^c^	87^b^	136^a^	74	98	72	87	89	90	96	± 51.1
K	< 0.001	0.010	0.985	185^a^	155^b^	118^c^	196^a^	136^b^	161^a^	150^b^	153^b^	134^b^	137^b^	± 51.9
Cl	0.001	0.404	0.959	129^b^	155^b^	186^a^	176	153	170	168	140	135	156	± 64.8
Mg	0.831	0.380	0.801	8.43	7.71	8.28	8.48	7.56	7.88	6.19	10.53	7.20	9.16	± 5.77
NH_4_	0.183	0.075	0.853	15.8	11.9	10.4	12.5	9.2	10.9	11.6	7.2	17.9	19.9	± 13.8
Crea	0.835	0.240	0.198	22.5	20.7	20.5	20.3	30.0	24.5	17.7	16.2	19.3	20.7	± 17.0
NABE	0.021	0.503	0.180	42.8^a^	21.0^b^	18.0^b^	24.9	32.6	14.0	32.3	41.1	15.4	30.7	± 44.0
Acids	0.034	0.159	0.462	79.0^a^	73.7^a^^b^	60.0^b^	69.6	67.3	89.7	62.6	64.0	79.5	63.7	± 34.0
Bases	< 0.001	0.981	0.512	138^a^	107^b^	88^c^	107	108	114	106	113	113	117	± 39.7
BSQ	0.731	0.679	0.324	1.6	1.7	1.5	1.6	1.8	1.3	1.8	1.8	1.6	1.3	± 1.17
Volume	0.043	0.486	0.915	13.8^b^	16.7^a^^b^	21.3^a^	14.4	14.2	14.0	19.7	21.2	17.5	19.7	± 13.1

bases in mmoL/L; minerals in mmol/L; NABE, net acid base excretion in mmol/L; estimated urine volume in L/d

^abc^ with *P* < 0.05 significant differences between means within ‘variant’ or ‘time’.

### Digestibility of proximate nutrients and minerals

**Fig 1:**
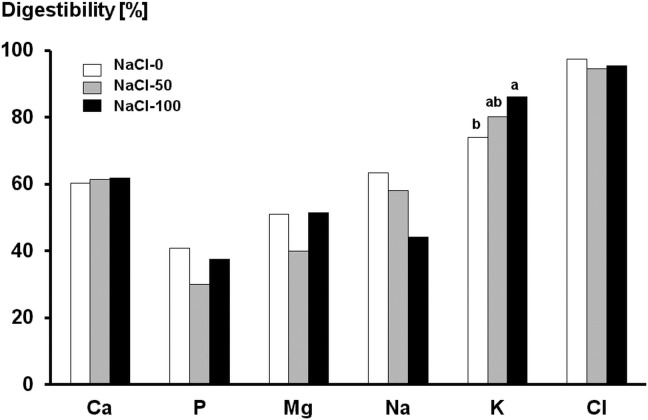
Mean apparent digestibility [%] of minerals in six horses receiving a core diet either without NaCl (NaCl-0) or added with 50 and 100 g of NaCl per day, respectively. (± pooled SD for digestibility of Ca, P, Mg, Na, K, and Cl = 2.36, 4.19, 11.7, 14.2, 6.80, and 3.01;^ab^ unequal superscripts indicate with *P* < 0.05 different means).

The mean digestibility of crude protein, acid ether extract and neutral detergent fiber was 71.6 ± 5.15%, 46.8 ± 13.6%, and 49.7 ± 9.83%, respectively, with no effect of diet. Means ± pooled standard deviation (SD) for digestibility of major elements are given in Fig 1. The only significant effect of NaCl supplementation was on potassium digestibility, which increased when salt was fed (*P* < 0.05 for NaCl-100 *vs* NaCl-0). There was a trend (*P* = 0.064) for a decrease of sodium digestibility when 100 g of NaCl were given.

### Acid-base variables

100 g but not 50 g of NaCl supplementation significantly decreased blood pH and BE (*P* < 0.05; Table 3). There was a corresponding trend of blood HCO_3_^-^ (*P* = 0.071). In addition, the 100 g NaCl supplementation caused a significant decrease in urine pH, acids, bases and NABE excretion (Table 2). Although 50 g of NaCl did not have a significant effect on urine pH (*P* > 0.05) it caused a significant decrease in acids, bases and NABE_1_ (*P* < 0.05; Table 2).

**Table 3:** Acid-base variables in the total blood of six horses within the individual feeding variant and days of dietary treatment, respectively (mean ± pooled SD; *P* values from analysis of variance; for individual results see S1 Table).

	*P* value			Mean			Pooled SD						
	for variance-factor			diet [g NaCl/d]									
	diet(d)	time (t)	d * t	0	50	100							
pH	0.048	0.023	0.959	7.414^a^	7.412^a^^b^	7.406^b^			± 0.0				
pCO_2_	0.901	0.262	0.993	49.4	49.5	48.1			± 2.509				
HCO_3-_	0.071	0.001	0.946	30.9	30.8	30.2			± 1.539				
BE	0.028	< 0.001	0.924	5.1^a^	5.1^a^	4.4^b^			± 1.40				

pCO_2_ in mmHg, HCO_3_^-^ in mmol/L, BE in mmol/L

^ab^ with *P* < 0.05 significant differences between means within ‘variant’ or ‘time’.

According to analysis of variance (Table 3), blood pH value and BE were significantly affected by the day of the treatment and the dietary treatment, with no interaction between these factors (*P* > 0.05). The blood pH value and the BE decreased when 100 g of NaCl was added to the diet (*P* < 0.05) by 0.008 units and 0.7 mmol/L, respectively. However, there was no significant effect of 50 g of NaCl (*P* > 0.05).

### Blood and urine biochemical variables

#### Blood

Serum creatinine, total protein, calcium, sodium, potassium, chloride and inorganic phosphorus were not affected by the diet. However, diet did affect serum concentration of magnesium and the degree of Ca-ionization (Table 4). Serum magnesium was significantly lower when 100 g of NaCl were fed, but the difference was small and values remained within reference range. The degree of Ca-ionization was significantly higher with 50 g of NaCl compared to the other two dietary treatments.

**Table 4:** Biochemical variables in the blood serum of six horses within the individual feeding variants and days of dietary treatment, respectively (mean ± pooled SD; *P* values from analysis of variance; for individual results see S1 Table).

	*P* value			Mean										Pooled SD
	for variance-factor			diet [g NaCl/d]			time [day of treatment]							
	diet d	time t	d * t	0	50	100	0	1	2	3	4	8	15	
Ca	0.387	0.026	0.989	2.60	2.56	2.60	2.51	2.51	2.55	2.60	2.64	2.64	2.63	± 0.155
Ca^++^	0.147	0.189	0.974	1.51	1.53	1.50	1.50	1.53	1.47	1.52	1.53	1.53	1.53	± 0.078
Ca^++^%	0.018	0.348	0.966	58.4^b^	61.1^a^	58.0^b^	60.4	60.1	57.3	58.2	58.5	58.8	60.9	± 4.78
Na	0.776	0.001	0.294	137	136	137	141^a^	137^b^	138^b^	137^b^	134^b^	136^b^	135^b^	± 4.46
K	0.137	0.373	0.454	3.6	3.6	3.7	3.7	3.7	3.7	3.6	3.7	3.6	3.5	± 0.27
Cl	0.784	<0.001	0.122	102	102	102	105^a^	102^b^^c^	103^a^^b^	101^b^^c^	100^c^	102^b^^c^	101^b^^c^	± 3.42
Mg	0.049	0.789	1.000	0.66^a^	0.64^a^^b^	0.63^b^	0.64	0.65	0.65	0.64	0.63	0.64	0.66	± 0.055
P_i_	0.339	<0.001	0.653	1.11	1.05	1.06	1.16^a^^b^	1.03^b^^c^	0.90^c^	1.02^b^^c^	1.10^b^	1.26^a^	1.07^b^	± 0.202
Crea	0.242	0.255	0.853	125	128	120	121	130	129	122	118	119	130	± 19.9
TP	0.973	0.003	1.000	61.3	61.9	61.4	64.6^a^^b^	54.6^b^	56.1^b^	57.9^b^	59.5^a^^b^	67.2^a^^b^	70.9^a^	± 13.7

Ca, Ca^++^, Na, K, Cl, Mg, and P_i_ in mmol/L; TP in g/L; Ca^++^%, degree of Ca-ionization in %

^abc^ with *P* < 0.05 significant differences between means within ‘variant’ or ‘time’.

#### Urine

Increasing the amount of NaCl supplemented significantly increased the concentrations of sodium and chloride in the urine while that of potassium decreased (*P* < 0.001; Table 2). There was no dietary effect on the overall concentration of the other minerals. There was, however, a time effect on the urinary calcium concentration with significantly higher levels being found at the end of the trials (day 15). This pattern was seen in all three diets but was most pronounced in the diet with 50 g NaCl.

#### Fractional clearance

Increasing the intake of NaCl elevated the FC of sodium and chloride considerably (Table 5). With NaCl-100, the FC of sodium and chloride was nearly five times and over twice, respectively, of the values found with NaCl-0. The FC of phosphorus was influenced by the day of treatment (*P* = 0.017) with particularly low and high percentages, respectively, at d8 and d15. However, there was neither a significant time-effect nor an interaction between the dietary treatment and the day of treatment for the FC of Ca, Mg, Na, K, and Cl (*P* > 0.05).

**Table 5:** Fractional renal clearance (FC) of minerals in six horses within the individual feeding variants and days of dietary treatment, respectively (mean ± pooled SD; *P* values from analysis of variance; for individual results see S1 Table).

	*P* value			Mean										Pooled SD
	for variance-factor			diet [g NaCl/d]			time [day of treatment]							
FC	diet(d)	time (t)	d * t	0	50	100	0	1	2	3	4	8	15	
Ca	0.284	0.086	0.508	4.71	6.54	5.24	5.72	5.69	8.01	3.36	3.13	5.70	6.90	± 5.41
Na	< 0.001	0.376	0.975	0.239^c^	0.664^b^	1.162^a^	0.447	0.500	0.422	0.846	0.931	0.691	0.980	± 0.961
K	0.145	0.338	0.992	30.0	31.9	26.0	32.6	24.2	29.7	33.0	32.8	24.7	30.1	± 13.9
Cl	< 0.001	0.538	0.923	0.82^b^	1.23^b^	1.72^a^	1.13	1.00	1.19	1.48	1.39	1.07	1.53	± 0.971
Mg	0.434	0.231	0.492	7.33	8.38	8.22	8.28	6.92	8.23	6.78	9.15	7.02	9.47	± 3.97
P_i_	0.732	0.017	0.611	0.366	0.387	0.416	0.361^a^^b^	0.329^a^^b^	0.500^a^^b^	0.347^a^^b^	0.317^a^^b^	0.282^b^	0.594^a^	± 0.292

Ca, Na, K, Cl, Mg, and P_i_ in %

^abc^ with *P* < 0.05 significant differences between means within ‘variant’ or ‘time’.

## Discussion

The quantity of ME supplied *via* the core diet and the horses’ workload corresponded very well with each other indicated by the approximate constancy of the horses’ bwt during the study.

The addition of 100 g of NaCl to a 60:40 hay:concentrate diet, with adequate intakes of calcium, magnesium and phosphorus, fed to horses in moderate work resulted in a mild metabolic acidosis. 50 g of NaCl supplementation under the same conditions appeared to result in a compensated metabolic acidosis as the urine and blood pH were not significantly affected. However, there was still an effect on NABE fractions. It is important to note that although the changes were statistically significant there were no apparent clinical consequences under these circumstances. Short-term mild compensated acidosis does not present a problem in itself. From the present study, we cannot predict long-term consequences. It is, however, worthwile to speculate on potential impact of other factors affecting acid-base balance when combined with an already existing compensated acidosis. Such factors include exercise induced acidosis, very low forage intakes, dietary mineral imbalances, other acidifying agents being present. Furthermore, it might become more important in young horses with protracted bone formation. The present study confirms the results from studies with different study protocols, e.g. previous studies had an even shorter period of time [28], [30] or were based on an all-hay diet [29]. In contrast to these previous studies, however, in the present investigation the acid base status was assessed ^~^12 hours after the intake of a dose of NaCl, and the effect was studied over a longer 2 week feeding period. To our knowledge this is the first study which describes a persistent acidifying effect of supplemental NaCl.

This acidifying effect is very unlikely to be due to differences in the DCAD of the trial rations because these were very similar (maximum DCAD-difference: 11 mmol/kg DM). The DCAD calculation applied here, however, did not take into account differences in absorption rates of ions. It appears that the acidifying effect of NaCl is independent of DCAB and may be mediated by the absorption rate of sodium actually being substantially lower than that of chloride (Fig 1). Acidifying effects of neutral salts such as CaCl_2_, as a consequence of a lower absorption rate of the cation than the anion, have been described in cats, sows and horses [28], [47], [48], [29], [30]. The relatively low apparent digestibility of sodium is not a unique feature of the present study. It is in good agreement with data from a meta-analysis of the literature [20]. An acidifying effect of NaCl has previously been described in human beings [49], [50], [51], [52], [31].

In addition, even when bypassing the gut barrier and therefore the different absorption rates of the minerals in question, the intravenous infusion of substantial amounts of NaCl has been shown to induce acidosis in healthy human volunteers [53]. It is particularly interesting, however, that in the recent study the NaCl induced effects on acid base metabolism were for some analytes (NABE, fractionated net acid base excretion) more pronounced with 50 g of NaCl compared to control feeding than when comparing 100 *vs* 50 g of NaCl intakes. This suggests that there is no linear increase in the Fig 1 effects observed from the zero-intake to 50 g and then to 100 g of NaCl per horse and day. This finding is consistent with previous work by Schwarzer [28]. It is possible that this effect could be caused by a sodium storage mechanism being present, which was postulated for humans in the context of orthostatic hypotension. Apparently, in humans, tissues were able to remove expendable sodium through a sodium/hydrogen exchange on glucosaminoglycans [24] with the consequence that the physiological process of counteracting low blood pressure interrupts. Such a storage mechanism may have a threshold for activation. The non-linear effects of the two sodium doses in the present studies could then perhaps, at least in part, be explained by the high dose of sodium activating the storage system whereas the lower one does not.

**Fig 2:**
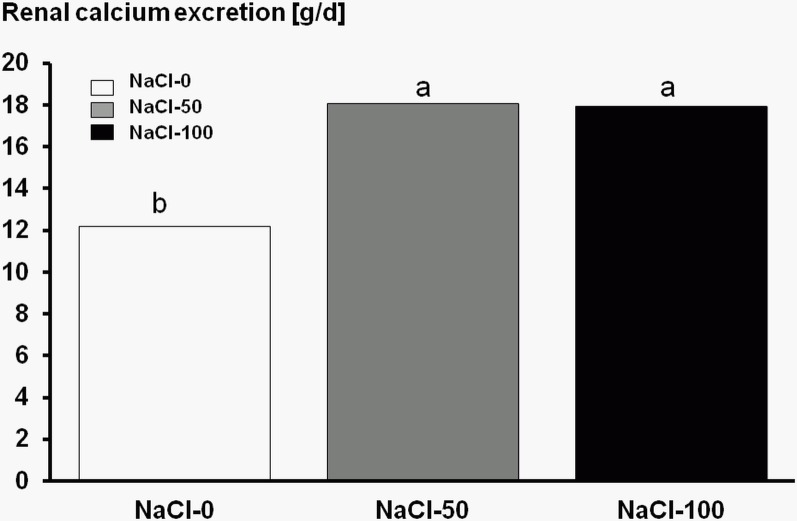
Mean apparent renal calcium excretion [g/d] in six horses receiving a core diet either without NaCl (NaCl-0) or added with 50 and 100 g of NaCl per day, respectively. (± pooled SD 2.17; ^ab^ unequal superscripts indicate with *P* < 0.05 different means).

It has been suggested that acidifying diets can result in the mobilisation of calcium from the bone in exercising horses [54]. Although in the present study there was no apparent effect of the NaCl supplementation and subsequent acidosis on the fractional renal excretion of calcium and magnesium, there was an effect of both doses of NaCl on estimated renal calcium excretion (Fig 2). At present, the clinical importance of this observation is not clear. Weanling horses consuming highly anionic diets and excreting more calcium *via* the urine did not show adverse effect on growth [23]. On the other hand, in Thoroughbred and Quarter racehorses, however, elevated intake of NaCl was associated with increased bone remodelling and reduced bone density [55]. In situations when more calcium is being deposited into bone such as periods of intense growth or increasing exercise intensity, elevated renal losses might be injurious particularly when the associated elevated requirement is not compensated by higher calcium intake.

The quantity of hay fed to the horses in the recent study is considered to be sufficient to reduce the risk of disturbances in hindgut fermentation [56]. It is also in agreement with feeding practices of many race and sports horses. For animal welfare reasons, however, more forage is recommended [57], [10].

Previous work has suggested that, higher intakes of hay may mask even the effect of strong acidifiers such as ammonium chloride [36]. It is therefore likely that higher forage intakes than in the present study may reduce or even eliminate the acidifying effects of any excess supplementary NaCl. It may also counteract any potential adverse effects on gastric mucosa.

Finally, although this study did show that NaCl supplementation can result in a mild metabolic acidosis, further work is required in order to evaluate the clinical relevance of this effect in particular any long term effects on calcium balance especially in the young growing, heavily exercising, animal.

The present study was carried out to look at the effect of NaCl on acid-base metabolism. The level of work, the corresponding sodium requirements according to NRC [2] or GfE ([58], [1]) and the sodium intake of the horses can also be used to comment upon the current recommended intakes of sodium. The horses in the present study were fed 1.4times maintenance energy requirements, and they maintained body weight. This suggests that their workload was correctly classified as moderate exercise according to NRC [2]. The sodium requirement of a 600 kg horse at moderate exercise level is estimated by both NRC [2] and GfE [1] to amount to about 20 g. The addition of 20 g sodium (NaCl 50) to a ration already providing 13 g sodium resulted in an intake of 33 g, i.e. 150% of sodium requirements. Therefore, it is not surprising that renal clearance of sodium increased even at this level of supplementation. The former recommendations for sodium supply for moderate work of GfE [58], however, were considerably higher (43 g sodium for a 600 kg horse and moderate work) than the intake in the present study. The results of the present study suggest that the reduction of the recommendations of GfE [1] for sodium supply to a similar figure as recommended by the NRC [2] was justified. Even on the basal diet with a sodium intake of only 13 g, compared to a recommended intake of 20 g, there was a considerable sodium content in urine. Since horses can down-regulate renal sodium excretion to very low values if they do not ingest sufficient sodium [11] the excretion of considerable amounts of sodium by urine suggests that sodium requirements of horses in moderate work may be even lower than the NRC [2] and GfE [1] recommendations–at least under similar conditions as in the present study (i.e. cool environmental temperature).

In principle the same is true for chloride, even though the recommendations for chloride requirements are not entirely based on factorial calculations. The intake with the unsupplemented basal diet amounted to 57 g compared to a recommended intake of 98 g [58], 64 g [2], and only 33 g [1]. There was a considerable chloride content in urine of the unsupplemented horses although horses can effectively down-regulate renal chloride excretion in case of low intake [11]. These results suggest that the reduction of the recommendations for chloride intake for working horses of GfE [1] was justified.

## Supporting Information

S1 Table Biochemical variables in blood and urine of the individual horses. (PDF)

## References

[1] GfE (2014) Energie- und Nährstoffbedarf landwirtschaftlicher Nutztiere. Nr. 11. Empfehlungen zur Energie- und Nährstoffversorgung von Pferden (Hrsg, Ausschuss für Bedarfsnormen der Gesellschaft für Ernährungsphysiologie). Frankfurt/Main, DLG Verlag

[2] NRC (2007) Nutrient requirements of horses. 6^th^ rev. ed. (ed., National Research Council). Washington, DC, National Academy Press

[3] Snow DH, Kerr MG, Nimmo MA, Abbott EM (1982) Alterations in blood, sweat, urine and muscle composition during prolonged exercise in the horse. Vet Res 110:377–384 10.1136/vr.110.16.377 7080430

[4] Kerr MG, Snow HD (1983) Composition of sweat of the horse during prolonged epinephrine (adrenaline) infusion, head exposure and exercise. Am J Vet Res 44:1571–1577 6625308

[5] McCutcheon LJ, Geor RJ, Hare MJ, Kingston JK, Staemfli HR (1995b): Sweat composition: comparison of collection methods and effects of exercise intensity. Equine Vet J, Suppl 18:279–284

[6] Meyer H (1987) Nutrition of the equine athlete. Equine Exercise Physiol II. ICEEP Publ:644–673

[7] Coenen M (1991) Chloridhaushalt und Chloridbedarf des Pferdes. Stiftung Tierärztliche Hochschule Hannover: Habilitation thesis

[8] McCutcheon LJ, Geor RJ (1998) Sweating—Fluid and ion losses and replacement. Veterinary Clinics of North America–Equine Practice 14(1):75–82 10.1016/s0749-0739(17)30213-4 9561689

[9] Jose-Cunilleras E (2004) Abnormalities of body fluids and electrolytes in athletic horses In: Equine sports medicine and surgery (eds., Hinchcliff KW, Kaneps AJ, Geor RJ). London, Elsevier:898–917

[10] Harris PA, Schott HC II (2013) Nutritional management of the elite endurance horses In: Equine Applied and Clinical Nutrition (eds., Geor RJ, Harris PA, Coenen M). Saunders Elsevier:261–270

[11] Lindner A, Schmidt M, Meyer H (1983) Investigations on sodium metabolism in exercised Shetland ponies fed a diet marginal in sodium In: Equine exercise physiology (eds., Snow DH, Persson SGB, Rose R.J). Cambridge, Burlington Press:310–317

[12] Jansson A, Dahlborn K (1999) Effects of feeding frequency and voluntary salt intake on fluid and electrolyte regulation in athletic horses. J Appl Physiol 86:1610–1616 10233125 10.1152/jappl.1999.86.5.1610

[13] Brunner J, Wichert B, Burger D, von Peinen K, Liesegang A (2012) A survey on the feeding of eventing horses during competition. J Anim Physiol Anim Nutr 96:878–884 10.1111/j.1439-0396.2012.01324.x 22809115

[14] Kennedy MAP, Entrekin P, Harris PA, Pagan JD (1998) Voluntary intake of loose versus block salt and its effect on water intake in mature idle Thoroughbreds. Equine Nutr Conf Feed Manufacturers:73–75

[15] Smith F (1890) Note on the composition of the sweat of the horse. J Physiol 11:497–503 16991935 10.1113/jphysiol.1890.sp000350 PMC1514200

[16] McCutcheon LJ, Geor RJ, Hare MJ, Ecker GL, Lindinger MI (1995a) Sweating rate and sweat composition during exercise and recovery in ambient heat and humidity. Equine Vet J, Suppl 20:153–157 10.1111/j.2042-3306.1995.tb05022.x 8933099

[17] McCutcheon LJ, Geor RJ, Ecker GL, Lindinger MI (1999) Equine sweating responses to submaximal exercise during 21 days of heat acclimation. J Appl Physiol 87(5):1843–1851 10562629 10.1152/jappl.1999.87.5.1843

[18] Spooner HS, Nielsen BD, Schott HC, Harris PA (2010) Sweat composition in Arabian horses performing endurance exercise on forage-based, low Na rations. Equine Vet J 42: 382–386 10.1111/j.2042-3306.2010.00208.x 21059034

[19] Zeyner A, Romanowski K, Vernunft A, Harris P, Kienzle E (2014) Scoring of sweat losses in exercised horses–a pilot study. J Anim Physiol Anim Nutr 98:246–250 10.1111/jpn.12073 23534876

[20] Kienzle E, Burger A. (2011) Maintenance requirements of macro-elements in horses. Übers Tierernährg 39:67–104

[21] Denton D. (1982): The consummatory act of satiation of salt appetite in sodium deficiency The hunger for salt. Berlin, Springer:278

[22] Nachman M, Valentino DA (1966) The role of taste and post-ingestional factors in the satiation of sodium appetite in rats. J Comp Physiol Psychol 62:280–283 5969606 10.1037/h0023667

[23] Cooper SR, Topliff DR, Freeman DW, Breazile JE, Geisert RD (2000) Effect of dietary cation-anion difference on mineral balance, serum osteocalcin concentration and growth in weanling horses. J Equine Vet Sci 20(1):39–44

[24] Gerzer R, Heer M (2005) Regulation of body fluid and salt homeostasis–from observations in space to new concepts on Earth. Curr Pharm Biotechnol 6:299–304 16101468 10.2174/1389201054553662

[25] Donovan DS, Solomon CG, Seely EW, Williams GH, Simonson DC (1993) Effect of sodium intake on insulin sensitivity. Am J Physiol 264:E730–E734 8498495 10.1152/ajpendo.1993.264.5.E730

[26] Tsugane S (2005) Salt, salted food intake, and risk of gastric cancer: Epidemiologic evidence. Cancer Sci 96:1–6 doi: 10.1111/j.1349-7006.2005.00006.x 15649247 10.1111/j.1349-7006.2005.00006.x PMC11158463

[27] Holbrook TC, Simmons RD, Payton ME, MacAllister CG (2005) Effect of repeated oral administration of hypertonic electrolyte solution on equine gastric mucosa. Equine Vet J 37:501–504 16295925 10.2746/042516405775314880

[28] Schwarzer U (1997) Untersuchungen zum Säure-Basen- und Elektrolythaushalt bei Stuten unter besonderer Berücksichtigung der Säuren- und Basenausscheidung im Harn. Universitäty of Leipzig, Doctoral thesis

[29] Stürmer K (2005) Untersuchungen zum Einfluss der Fütterung auf den Säure-Basen-Haushalt bei Ponys. München: Ludwig-Maximilians-Universität, Doctoral thesis

[30] Zeyner A, Schwarzer U, Fürll M (2005) Effects of an oral sodium chloride load at different levels on acid-base balance and renal mineral excretion in horses. Proc 9th Congr ESVCN, Grugliasco (Italy):124

[31] Frings-Meuthen P, Baecker N, Herr M (2008) Low-grade metabolic acidosis may be the cause of sodium chloride-induced exaggerated bone resorption. J Bone Mineral Res 23:517–524 10.1359/jbmr.071118 18052757

[32] Berchtold L (2009) Untersuchungen zum Einfluss der Kationen-Anionen-Bilanz auf den Mineralstoff- und Säure-Basen-Haushalt von Ponys. Ludwig Maximilians University München, Doctoral thesis

[33] Zeyner A, Kirbach H, Fürll M (2002) Effects of substituting starch with fat on the acid-base and mineral status of female horses. Equine Vet J, Suppl 34:85–91 12405665 10.1111/j.2042-3306.2002.tb05397.x

[34] Kienzle E, Zeyner A (2010) The development of a metabolisable energy system for horses. J Anim Physiol Anim Nutr 94:e231–e240 10.1111/j.1439-0396.2010.01015.x 20626500

[35] Kienzle E, Coenen M, Zeyner A (2010) Maintenance matabolisable energy requirements in horses. Übers Tierernähr 38:33–54

[36] Kienzle E, Stürmer K, Ranz D, Clauss M (2006) A high roughage/concentrate ratio decreases the effect of ammonium chloride on acid-base balance in horses. J Nutr 136:2048S–2049S 16772493 10.1093/jn/136.7.2048S

[37] Goren G, Fritz J, Dillitzer N, Hipp B, Kienzle E (2014) Fresh and preserved green fodder modify effects of urinary acidifiers on urine pH of horses. J Anim Physiol Anim Nutr:98, 239–245 10.1111/jpn.12071 23551706

[38] Nehring K, Beyner M, Hoffmann B (1972) Futtermitteltabellenwerk (Hrsg.: Oskar-Kellner-Institut für Tierernährung des Forschungszentrums für Tierproduktion Dummerstorf-Rostock). 2. Auflage Berlin, VEB Deutscher Landwirtschaftsverlag

[39] McCarthy JF, Aherne FX, Okai DB (1974) Use of HCl-insoluble ash as an index material for determining apparent digestibility with pigs. Can J Anim Sci 54:107

[40] Schurg WA, Frei DL, Cheek PR, Holtan DW (1977) Utilization of whole corn plant pellets by horses and rabbits. J Anim Sci 45:1317–1321

[41] Sutton EI, Bowland JP, McCarthy JF (1977) Studies with horses comparing 4N-HCl insoluble ash as an index material with total fecal collection in the determination of apparent digestibilities. Can J Anim Sci 57:543–549

[42] Fuchs R, Militz H, Hoffmann M (1987) Untersuchungen zur Verdaulichkeit der Rohnährstoffe bei Pferden. 1. Mitteilung: Methoden zur Bestimmung der Verdaulichkeit. Arch Anim Nutr 37:235–246 10.1080/17450398709428239 3689141

[43] VDLUFA (1976) Verband Deutscher Landwirtschaftlicher Untersuchungs- und Forschungsanstalten (Hrsg.). In: Handbuch der Landwirtschaftlichen Versuchs- und Untersuchungsmethodik (VDLUFA-Methodenbuch), Bd. III Die chemische Untersuchung von Futtermitteln. Loseblattsammlung mit Ergänzungen von 1983, 1988 und 1993. VDLUFA-Verlag, Darmstadt

[44] Fürll M., Garlt C., Lippmann R. (1981): Klinische Labordiagnostik. S. Hirzel-Verlag Leipzig.

[45] Meyer H, Stadermann B (1990) Assessing of the mineral supply of horses by urine analysis. Advances Anim Physiol Anim Nutr 21:86–97

[46] Harris PA, Gray J (1992) The use of the urinary fractional electrolyte excretion test to assess electrolyte status in the horse. Equine Vet Educ 4(4):162–166

[47] Kienzle E, Thielen C, Janowicz P (1998) Effect of urinary acidification using ammonium chloride on renal magnesium excretion in cats. J Anim Physiol Anim Nutr 80:130–133

[48] Beker S. (1999): Einstellung des Harn-pH-Wertes bei Sauen. Ludwig Maximilians University München, Doctoral thesis.

[49] Lucas PA, Lacour B, Comte L, McCarron DA, Drueke T (1988) Abnormal parameters of acid-base balance in genetic hypertension. Kidney Int Suppl 25:S19–S22 3184610

[50] Sharma AM, Kribben A, Schattenfroh S, Cetto C, Distler A (1990) Salt sensitivity in humans is associated with abnormal acid-base regulation. Hypertension 16:407–413 2210808 10.1161/01.hyp.16.4.407

[51] Sharma AM, Distler A (1991) Abnormal acid-base regulation in salt-sensitive normotensive man. Kli. Wschr 69 (Suppl 25):41–44 1921251

[52] Sharma AM, Distler A (1994) Acid-base abnormalities in hypertension. Am J Med Sci 307 (Suppl. 1):S112–S115 8141148

[53] Williams EL, Hildebrand KL, McCormick SA, Bedel MJ (1999) The effect of intravenous lactated Ringer's solution versus 0.9% sodium chloride solution on serum osmolality in human volunteers. Anesthesia & Analgesia 88(5):999–1003 10320158 10.1097/00000539-199905000-00006

[54] Wall DL, Topliff DR, Freeman DW, Wagner DG, Breazile JW, Stutz WA (1992) Effect of dietary cation-anion balance on urinary mineral excretion in exercised horses. J Equine Vet Sci 12(3):168–171

[55] MacLeay JM, Mayo JM (2006) Effect of dietary acid load on serum osteocalcin in Thoroughbred and Quarter racehorses. Proceedings of 7th International Conference on Equine Exercise Physiology; 26–31.08.2006; Fontainebleau, (France) p. 210.

[56] Zeyner A, Geißler C, Dittrich A (2004) Effects of hay intake and feeding sequence on variables in faeces and faecal water (dry matter, pH value, organic acids, ammonia, buffering capacity) of horses. J. Anim. Physiol. Anim. Nutr. 88:7–19 10.1111/j.1439-0396.2004.00447.x 19774758

[57] Zeyner A, Kienzle E, Coenen M (2011) Artgerechte Pferdefütterung. Pferdezucht, -haltung und–fütterung. Landbauforschung Völkenrode, Sonderheft 353:164–191

[58] GfE (1994): Energie- und Nährstoffbedarf landwirtschaftlicher Nutztiere Nr. 2 Empfehlungen zur Energie- und Nährstoffversorgung der Pferde (Hrsg.: Ausschuss für Bedarfsnormen der Gesellschaft für Ernährungsphysiologie). Frankfurt/Main: DLG Verlag.

