# Supplementary material for: Effects of Different Oral Doses of Sodium Chloride on the Basal Acid-Base and Mineral Status of Exercising Horses Fed Low Amounts of Hay
Source: PLoS One. 2017 Jan 3;12(1):e0168325. doi: 10.1371/journal.pone.0168325 (PMC5207637; doi:10.1371/journal.pone.0168325)
Supplement: S1 Table — (PDF) [file pone.0168325.s001.pdf]

NaCl intake<br>0 - NaCl-0<br>1 - NaCl-50<br>2 - NaCl-100	day of<br>treatment	number of<br>the horse	number of<br>the trial<br>period			
**NaCl**	**day**	**horse**	**period**	pH	pCO2	HCO3
0	0	1	1	7,439	45,6	30,2
0	2	1	1	7,445	49,2	33
0	3	1	1	7,401	50,2	30,4
0	4	1	1	7,439	50,6	33,5
0	5	1	1	7,425	47,9	30,7
0	9	1	1	7,392	50,1	29,7
0	16	1	1	7,431	48,6	31,6
1	0	1	2	7,413	52,8	32,9
1	2	1	2	7,401	52,7	32
1	3	1	2	7,39	52,3	30,9
1	4	1	2	7,415	52,5	32,9
1	5	1	2	7,43	49,9	32,4
1	9	1	2	7,418	50,9	32,1
1	16	1	2	7,408	51	31,5
2	0	1	3	7,408	51	31,4
2	2	1	3	7,399	49,1	29,7
2	3	1	3	7,341	50,4	26,7
2	4	1	3	7,397	49,1	29,5
2	5	1	3	7,4	49	29,7
2	9	1	3	7,416	49,4	31
2	16	1	3	7,414	47,4	29,6
0	0	2	1	7,419	43,4	27,4
0	2	2	1	7,42	49,8	31,6
0	3	2	1	7,422	48,1	30,7
0	4	2	1	7,446	47,2	31,8
0	5	2	1	7,415	48,6	30,5
0	9	2	1	7,416	46,8	29,4
0	16	2	1	7,431	46,7	30,4
1	0	2	2	7,421	49	31,1
1	2	2	2	7,396	49,9	30
1	3	2	2	7,403	46	28
1	4	2	2	7,417	48,3	30,4
1	5	2	2	7,42	47,7	30,3
1	9	2	2	7,411	48,3	30
1	16	2	2	7,427	46,8	30,2
2	0	2	3	7,419	47,4	30
2	2	2	3	7,391	49,3	29,3
2	3	2	3	7,375	45,8	26,2
2	4	2	3	7,416	47,2	29,7
2	5	2	3	7,424	48,6	31,1
2	9	2	3	7,424	47	30,1
2	16	2	3	7,406	48,5	29,8
0	0	3	1	7,413	49,9	31,1

0	2	3	1	7,4	51,9	31,4
0	3	3	1	7,376	49,6	28,5
0	4	3	1	7,421	51,9	33
0	5	3	1	7,422	51,5	32,8
0	9	3	1	7,447	46,8	31,6
0	16	3	1	7,425	49,7	31,8
1	0	3	2	7,394	48,9	29,2
1	2	3	2	7,435	50,1	32,9
1	3	3	2	7,442	49,9	33,3
1	4	3	2	7,422	54,3	34,6
1	5	3	2	7,421	52,1	33,1
1	9	3	2	7,415	52,8	33,1
1	16	3	2	7,423	53,8	34,4
2	0	3	3	7,404	7,413	31,1
2	2	3	3	7,376	53,7	30,7
2	3	3	3	7,406	47,8	29,4
2	4	3	3	7,423	53,7	34,3
2	5	3	3	7,407	53,8	33,1
2	9	3	3	7,403	54,1	33
2	16	3	3	7,398	53,3	32,1
0	0	4	3	7,402	50,8	30,9
0	2	4	3	7,406	51,1	31,3
0	3	4	3	7,381	51	29,6
0	4	4	3	7,408	50,7	31,3
0	5	4	3	7,391	51,4	30,5
0	9	4	3	7,4	50,6	30,6
0	16	4	3	7,413	49,6	30,9
1	0	4	1	7,39	46,9	27,7
1	2	4	1	7,413	51,1	31,8
1	3	4	1	7,419	49,5	31,3
1	4	4	1	7,408	50	30,9
1	5	4	1	7,41	49,5	30,7
1	9	4	1	7,407	50,2	30,9
1	16	4	1	7,415	48,6	30,5
2	0	4	2	7,406	49,3	30,3
2	2	4	2	7,372	50,7	28,8
2	3	4	2	7,416	45	28,3
2	4	4	2	7,416	49,2	30,9
2	5	4	2	7,4	50,8	30,8
2	9	4	2	7,403	51,7	31,5
2	16	4	2	7,386	48,9	28,6
0	0	5	2	7,401	52,1	31,6
0	2	5	2	7,387	53,3	31,4
0	3	5	2	7,406	48,9	30
0	4	5	2	7,417	50,3	31,6
0	5	5	2	7,406	53,3	32,7
0	9	5	2	7,417	53,2	33,5
0	16	5	2	7,401	52,2	31,7
1	0	5	3	7,421	49,8	31,7
1	2	5	3	7,404	47,8	29,2

1	3	5	3	7,377	51,7	29,7
1	4	5	3	7,39	50	29,6
1	5	5	3	7,418	48,9	30,9
1	9	5	3	7,417	49,5	31,2
1	16	5	3	7,387	47,7	28
2	0	5	1	7,41	45,1	27,9
2	2	5	1	7,43	48,5	31,5
2	3	5	1	7,414	49,1	30,7
2	4	5	1	7,412	50,1	31,2
2	5	5	1	7,418	50,6	31,9
2	9	5	1	7,408	48,7	30,1
2	16	5	1	7,42	52	33
0	0	6	2	7,405	48,3	29,6
0	2	6	2	7,392	48,8	29
0	3	6	2	7,438	43,6	28,8
0	4	6	2	7,419	49,6	31,4
0	5	6	2	7,431	45,2	29,4
0	9	6	2	7,419	49,1	31
0	16	6	2	7,406	48,6	29,8
1	0	6	3	7,415	47,6	29,8
1	2	6	3	7,407	50	30,7
1	3	6	3	7,4	44,4	26,9
1	4	6	3	7,408	47,2	29,1
1	5	6	3	7,426	45,8	29,4
1	9	6	3	7,432	46,7	30,5
1	16	6	3	7,424	44,8	28,7
2	0	6	1	7,406	44,5	27,4
2	2	6	1	7,419	45,7	28,9
2	3	6	1	7,394	47,1	28,2
2	4	6	1	7,425	47,9	30,7
2	5	6	1	7,417	46,1	29,1
2	9	6	1	7,408	50,4	31,1
2	16	6	1	7,414	47,1	29,4

parameters in blood

BE	Cl	TP	K	Ca	Crea	Mg
5,2	104	62	3,4	2,5	122	0,73
7,5	99	57	3,5	2,48	141	0,68
4,5	106	54,9	4	2,48	156	0,68
7,8	99	56,2	3,6	2,59	128	0,67
5,2	88	49,7	3,3	2,92	109	0,72
3,7	101	63,4	3,6	2,75	131	0,83
6,1	97	60	3,4	2,76	120	0,78
6,7	99	57,9	3,5	2,42	128	0,6
5,7	100	58,9	3,5	2,37	126	0,62
4,6	101	59,5	3,5	2,48	122	0,7
6,7	100	60	3	2,42	129	0,69
6,7	100	58,4	3,6	2,47	118	0,67
6,2	101	60,9	2,9	2,32	113	0,67
5,5	99	64,2	3,7	2,48	118	0,63
5,4	102	55,9	3,8	2,47	117	0,65
3,8	102	55,3	3,3	2,48	141	0,67
0,1	105	57,5	3,5	2,42	126	0,65
3,7	103	56,4	3,1	2,37	126	0,69
3,9	102	58	3,2	2,48	125	0,62
5,3	101	59,9	3,7	2,37	134	0,64
4,1	101	59,8	3,8	2,58	131	0,63
2,5	103	54,7	3,5	2,6	102	0,55
5,8	101	52,6	3,5	2,59	125	0,64
5,2	101	56,5	4	2,48	134	0,58
6,6	101	50,5	3,6	2,76	119	0,67
4,9	84	43,4	3,1	2,98	103	0,65
4	104	57,1	3,6	2,91	118	0,66
5,1	98	53,2	3,6	2,71	112	0,69
5,5	105	55,2	3,6	2,26	119	0,62
4	103	54,2	3,6	2,42	110	0,61
2,6	103	54,2	3,6	2,42	116	0,62
4,8	104	52,2	3,6	2,47	118	0,62
4,8	103	52,4	3,7	2,52	104	0,61
4,4	104	54,4	3,7	2,63	102	0,6
4,9	102	59	3,6	2,48	110	0,58
4,5	103	53,2	3,7	2,63	105	0,58
3,3	104	52,9	3,8	2,37	119	0,63
0,5	105	52	3,7	2,71	121	0,63
4,2	103	51,8	3,8	2,48	115	0,6
5,5	103	51,3	3,5	2,42	114	0,55
4,7	102	54,6	3,9	2,37	108	0,61
4,1	102	51,4	3,7	2,52	111	0,61
5,3	103	57,5	3,7	2,58	99	0,63

5,2	101	56,6	3,8	2,53	116	0,68
2,3	103	56	4	2,69	144	0,61
7	101	53,5	3,8	2,58	109	0,61
6,8	100	56,8	3,7	2,53	111	0,62
6,5	101	59,4	3,6	2,58	111	0,64
6,1	101	57	3,7	2,68	115	0,63
3,3	103	59,4	4	2,65	98	0,68
7,2	100	58,3	4	2,7	119	0,67
7,7	103	53,9	3,8	2,37	152	0,61
8,2	99	55,4	3,8	2,92	110	0,62
7,1	96	51,8	3,8	2,81	105	0,66
6,9	99	59,9	3,9	2,91	113	0,73
8,7	98	55,9	3,6	2,81	280	0,8
5,3	103	57,5	4,1	2,15	122	0,68
4,1	103	60,9	4,1	2,7	103	0,64
3,8	102	57,3	4	2,69	100	0,68
8	102	58,2	3,8	2,58	107	0,59
6,7	101	56,6	4	2,73	98	0,66
6,5	102	60,7	3,9	2,63	97	0,65
5,7	101	63,6	3,7	2,64	104	0,66
4,9	102	56,5	3,5	2,63	120	0,63
5,3	102	54,9	2,9	2,37	149	0,68
3,3	104	57,3	3,6	2,69	137	0,86
5,3	103	55,4	3,8	2,53	134	0,67
4,3	102	56	3,8	2,53	131	0,61
4,6	102	59	3,4	2,63	134	0,62
5,1	101	55,7	3,1	2,63	134	0,67
2,1	103	58,2	4	2,55	127	0,66
5,9	102	54,1	3,3	2,48	154	0,68
5,6	102	54,2	3,8	2,48	120	0,68
5	102	53,1	3,4	2,81	139	0,7
4,9	101	52,8	4	2,81	138	0,66
5	104	58	3,4	2,91	139	0,61
4,8	98	56,6	3	2,71	128	0,71
4,5	105	59,9	3,4	2,53	142	0,63
2,5	104	54,8	3,8	2,53	129	0,58
3,1	104	56,1	3,7	2,69	127	0,64
5,2	103	56,7	3,6	2,69	130	0,64
4,7	104	52,8	3,9	2,78	119	0,65
5,4	102	54,8	3,6	2,68	105	0,54
2,7	104	67	3,1	2,69	122	0,62
5,4	128	76,6	4,7	2,42	156	0,73
4,9	101	60,1	3,7	2,48	115	0,59
4,2	102	57,3	3,7	2,53	116	0,59
5,8	101	61,8	3,8	2,79	116	0,66
6,4	101	57,3	3,7	2,73	115	0,62
7,2	100	57,8	3,7	2,58	102	0,55
5,4	103	63,5	3,5	2,48	131	0,62
5,9	102	56,3	3,1	2,53	109	0,58
3,6	103	56,9	3,6	2,53	130	0,64

3,3	103	58,1	3,5	2,64	125	0,53
3,6	104	55,8	3,7	2,42	126	0,58
5,2	102	56,2	3,7	2,48	122	0,55
5,4	100	60,4	3,8	2,58	127	0,57
2,3	103	57	3,8	2,63	135	0,69
2,7	105	61,2	4,1	2,7	113	0,59
5,9	100	57,1	3,9	2,7	133	0,59
5	103	50,4	3,7	2,53	122	0,64
5,3	100	56,7	3,8	2,7	88	0,55
6	97	54,5	3,7	2,87	116	0,57
4,4	99	63,5	3,9	2,96	115	0,61
6,9	98	60,1	3,7	2,82	113	0,68
3,9	112	64,7	3,8	2,42	152	0,77
3,2	104	57,2	3,6	2,48	133	0,68
4	104	54,9	3,7	2,48	126	0,67
5,6	103	57,3	3,8	2,53	136	0,67
4,3	104	54,9	3,8	2,58	126	0,64
5,4	103	57,4	3,6	2,47	112	0,6
4,1	103	58,9	3,6	2,48	131	0,65
4,3	104	57	3,5	2,53	122	0,67
4,8	105	53,4	3,9	2,42	146	0,65
1,7	106	58,3	3,8	2,64	139	0,69
3,6	105	54	3,8	2,48	135	0,64
4,3	103	55	3,8	2,42	137	0,62
5,2	103	58,5	3,1	2,52	137	0,67
3,6	104	60,8	3,2	2,42	113	0,59
2,2	105	58,4	3,8	2,55	126	0,62
3,7	104	56,7	3,9	2,59	150	0,68
2,5	101	58	3,3	2,53	138	0,64
5,2	102	52,9	3,7	2,64	138	0,67
3,8	102	52,7	3,8	2,42	131	0,66
5,1	105	55,6	3,8	2,75	136	0,68
4	101	58,2	3,8	2,74	133	0,65

par

Na	P	NH4	bases	acids	Cl	density
137	0,91	4,5	145	32	163,5	1,038
134	1,14	10,5	175	114,5	139,8	1,048
138	0,84	15,5	220	125	141,3	1,042
135	1,17	5	155	47,5	92,6	1,02
121	1,21	5	160	80	119,9	1,032
134	1,18	30,5	120	105	72,6	1,04
132	0,89	9	280	100	112,3	1,04
133	0,96	5,5	165	85	85,7	1,034
136	0,95	7,5	155	77	108,8	1,03
138	0,94	12	120	107,5	139,8	1,038
134	1,1	7,5	165	87,5	104,9	1,036
136	1,18	5	180	102,5	134,8	1,03
138	0,89	10	95	122,5	150,3	1,03
136	1,32	2,5	80	107,5	155,5	1,032
136	0,97	60	55	57,5	284	1,03
136	1,03	5	85	90	195,5	1,03
139	0,84	10	100	130,5	120,9	1,03
138	1,09	10	50	70	105,8	1,03
137	1,18	5	90	85	70,5	1,03
137	1,19	4	65	67,5	108,1	1,03
141	1,17	25,5	85	67,5	177	1,03
137	1,05	36	105	72,5	220,6	1,03
135	1,25	13	140	75	168,9	1,04
135	0,75	12,5	155	142,5	198,2	1,04
136	0,94	42,5	105	70	129,7	1,03
111	0,74	15	135	110	157,2	1,04
135	2,52	44,5	95	177,5	76,9	1,034
131	0,9	22	180	110	72,3	1,04
140	1,17	10,5	115	67,5	153,3	1,034
137	0,95	12	115	50	150,3	1,024
137	1,13	9	95	27,5	132	1,02
137	0,98	22,5	140	63	194,9	1,03
136	1,11	20	105	68	183,4	1,022
137	1,04	22,5	100	82	192,8	1,028
135	1,07	5	100	78	190,1	1,03
136	0,95	10	95	72,5	300,5	1,03
136	0,99	7,5	65	27,5	132,5	1,01
137	0,85	12,5	50	47	193,5	1,028
136	1,12	8	45	50	166,6	1,011
137	1,11	7	110	20	157,7	1,024
136	1,34	5,5	55	27,5	212,6	1,03
136	1,08	30	55	52,5	281	1,03
137	1,24	10	91	72,5	210,7	1,032

136	0,94	12,5	155	80	100,1	1,03
137	0,83	20	100	91	170,8	1,03
137	1,04	5	60	30	165,4	1,02
137	1,27	2,5	105	32,5	88,8	1,01
137	1,1	2,5	75	25	85,9	1,012
136	1,14	9	115	17,5	104	1,02
138	1,05	2	125	17,5	141,2	1,016
137	1,1	1	30	2,5	73,8	1,039
138	0,71	6	175	175	187,7	1,03
137	1,09	1	95	17,5	82,3	1,02
130	0,97	2,5	80	30	103,8	1,009
135	1,45	1	80	37,5	96,5	1,018
134	0,79	5	120	40	137,2	1,019
150	1,2	7,5	100	67,5	139,1	1,028
138	0,89	7,5	55	40	229,6	1,028
138	0,91	2,5	90	42	172,1	1,02
138	0,98	4	130	32	154,4	1,02
137	1,05	3	75	27,5	170,1	1,012
138	0,94	2,5	80	17	160,6	1,014
136	1,22	1	80	25,5	121,1	1,012
136	1,16	6,5	91	62,5	195,2	1,03
137	1,04	10	155	80	141,5	1,04
138	1,06	17,5	140	115	181,4	1,042
138	1,07	11,5	135	85	242,2	1,04
136	1,17	12,5	150	72,5	152,4	1,042
135	1,28	10,5	120	87	178,5	1,04
136	1,21	60	165	69	174	1,05
138	1,36	5	95	85	264,8	1,03
136	1,14	13,5	105	85	248,4	1,04
137	0,84	7	85	95,5	316,7	1,04
136	0,87	15,5	85	70,5	291,9	1,044
134	1,07	5,5	95	68	206,4	1,04
136	0,91	15	95	192,5	86,5	1,041
132	0,93	7	100	86	247,6	1,03
139	1,26	10	95	125	188,8	1,04
138	1,06	7,5	75	75	241,8	1,034
138	0,97	7,5	115	83,5	230,4	1,04
138	1,07	10	95	64	356,8	1,04
137	1,24	10	145	107,5	226,6	1,04
136	1,13	7,5	145	37,5	209,8	1,03
138	1,61	0,2	50	80	226	1,03
173	1,51	12	120	92	85,3	1,04
138	0,94	5,5	105	80	63,6	1,03
139	0,87	5	80	55,5	84,6	1,03
137	0,88	12,5	150	117	90,4	1,034
138	1,15	8	110	52	21,9	1,03
137	1,21	7,5	200	81	11,1	1,03
138	1,04	1,4	40	10	56,7	1,032
138	1,41	10	110	57,2	55,6	1,03
138	0,87	12	70	57	83,1	1,03

138	0,9	7,5	100	57,5	81	1,022
138	0,95	8	120	135	186,2	1,03
138	1,03	4	55	30	78,5	1,02
136	1,23	5	145	47,5	103,9	1,02
136	1,13	75	77,5	76	12	1,04
140	0,93	2	85	17,5	109,3	1,026
136	0,95	6	100	55	140,5	1,02
136	0,77	3,5	105	72,5	103	1,03
136	0,96	5	80	62,5	126,5	1,02
132	1,04	6	110	86	125,3	1,02
135	1,72	5	35	14,5	84,8	1,015
135	0,86	5	160	72	101,4	1,024
148	1,26	12,5	145	117	167,1	1,04
137	0,98	11,5	130	85,5	129,2	1,036
138	1	18	145	62,5	126,1	1,032
137	1,12	22	170	34	101,9	1,038
136	1,23	12	140	75	160,5	1,034
137	1,2	77,5	205	129	110,5	1,042
137	1,36	4	245	47,5	158	1,03
138	1,25	12,5	115	102	193,6	1,038
138	1,29	10	95	53	161,2	1,03
137	1,12	20	100	102	163,6	1,03
137	1,05	9	50	53	204,8	1,028
137	1,22	5	60	52,5	178,3	1,028
137	1,13	38,5	230	110	269,6	1,04
136	0,62	44	80	34	184	1,032
139	1,15	8	85	50	212,4	1,02
136	1,11	12,5	140	84,5	247,1	1,04
137	0,8	10	85	82,5	310,7	1,032
136	0,83	9,5	85	37,5	232,9	1,03
134	0,83	1	120	52,5	190,1	1,03
136	1,22	32	90	70	211,2	1,03
132	1	52	100	74	292,2	1,023

rameters in urine

K	Ca	Crea	Mg	Na	pH	air humidity%
253,4	12	22,1	8,75	12	7,8	76,1
115,6	36	19,85	9,95	5	7,08	78,3
159,6	28	18,82	12,45	5	7,31	82,5
165,6	2,1	12,8	2,25	68	8,13	78,9
235,9	4,3	23,9	14,2	9	8,44	78,1
173,9	17,5	41,2	9,65	18	7,06	71,7
268,9	5,4	25,1	9,4	4	8,08	83,1
193	50	28,7	9,8	6	7,57	75,4
141,7	6,4	23,1	8,25	130	7,82	86,3
182,6	37,5	36,1	9,75	10	7,65	85,5
223,6	5,7	25,2	9,35	6	7,2	83,3
179,6	6	20,7	9	103	7,75	78,8
140,4	8,75	23,7	9,5	43	6,8	77,5
135,9	62	26,9	10,6	24	6,42	78,8
185,4	13	15	8,65	105	7,19	70,7
134,9	18	17,6	7,7	130	7,51	73,2
151,2	15	14,6	9,6	54	5,05	73,9
140,4	33	33,9	8,1	74	5,24	74,2
152,4	20	14,9	9,35	27	5,33	74,4
114	26	16,5	10,5	147	6,88	73,0
100	25	21,7	10,45	164	7,43	64,6
244,2	15	20,8	6,35	49	7,96	76,1
148	55	16,48	9,85	7	7,13	78,3
168	42	16,44	9,55	5	7,21	82,5
152,1	6	16,7	7,2	91	7,64	78,9
162,6	13,5	30,3	6,15	8	7,3	78,1
138,4	16	24,6	9,55	12	7,16	71,7
	29	29,8	9,6	14	7,05	83,1
192,5	26	22,6	9,2	69	7,83	75,4
102,5	4,5	14	5,8	164	7,77	86,3
162,8	5,9	19,1	5,3	113	7,58	85,5
228,6	15,5	19,9	9,45	22	7,3	83,3
128,7	11,5	12,6	7,55	161	7,54	78,8
108,1	35	12,2	7,4	154	7,74	77,5
135,5	58	18,9	9,2	64	6,12	78,8
205,3	15	18,7	7,25	130	7,75	70,7
43,2	2,8	5,6	0,75	151	7,61	73,2
97,8	11	11,7	1,15	177	6,6	73,9
56,5	10	9,5	2,7	149	6,59	74,2
87,9	6	9,1	3,95	202	7,02	74,4
186,4	35	18,5	9,8	21	6,44	73,0
112	79	18,3	10,3	174	6	64,6
230,8	5	18,8	4,75	51	7,73	

157,8	17	23,6	8,9	86	7,43	
161,5	15	19,1	5,25	36	6,82	
99,7	11	7,4	1,8	128	7,21	
60,9	1,5	4	0,75	126	7,46	
102,2	4	7,1	2,7	76	7,99	
82	7	10,3	7,3	144	6,71	
86,6	1,6	6,5	1,1	176	8,36	
16,7	2,5	15,9	1,85	108	7,95	
181,6	17	87,6	9,7	31	7,27	
39,3	1	3,1	0,8	136	8,23	
51,2		4	1,9	126		
51,1	45	6,2	0,25	138	8,56	
145,3		9,5	7,1	108	7,6	
132	26	16,2	8,4	81	7,72	
106	40	15,6	8,6	150	7,22	
89,1	8,5	11,6	5,85	177	6,75	
57,7	2	4,5	3,1	202	7,44	
44,6	1,9	3,7	2,35	188	7,36	
38,4	3	5,7	1,8	202	7,51	
29,7	2	1,6	11,5	161	7,5	
244,9	9,5	17,7	9,25	4	8,24	76,1
157,8	17	23,6	8,9	86	7,43	78,3
173	72,5	28,2	8,7	5	6,85	82,5
212,8	40	27	9	6	6,33	78,9
142,3	7,5	25,8	9,5	7	7,26	78,1
173,8	32	26,9	10,6	7	7,28	71,7
131	120	37,8	10,2	7	6,98	83,1
227,2	22	19	8,95	83	7,97	75,4
164	60	16,4	9,75	25	7,64	86,3
140,3	80	13,46	9,15	23	7,47	85,5
162,1	20	25	10,35	43	7,93	83,3
180,3	20	23,5	14,8	59	7,44	78,8
150,6	31	24,3	9,45	50	7,59	77,5
127,4	52	26,2	9,1	93	7,4	78,8
168,3	60	27,1	9,4	64	5,55	70,7
136,7	35	24,8	9,95	129	6,77	73,2
183,2	65	34,4	9,45	42	7,36	73,9
181,1	11	25,2	9,5	44	7,15	74,2
206,7	45,5	22,2	10,35	23	6,77	74,4
154,4	4	15,5	7,1	183	7,4	73,0
90,2	40	16,3	10,1	175	5,21	64,6
202,4	50	25,4	18	15	7,25	76,1
188,1	30	23,1	9,45	53	7,03	78,3
215,3	45	18,1	7,45	51	7,82	82,5
172,6	17,5	20	9,7	12	6,78	78,9
228,4	33	17,3	10,05	15	7,36	78,1
209,5	2,5	23	4,2	45	7,63	71,7
232,9	20	21,8	13,4	18	7,14	83,1
244	9,5	23,1	8,9	42	7,8	75,4
260	25	21	8	44	7,12	86,3

159,3	30	9,3	9,3	130	7,17	85,5
204,7	20	21,4	6,2	67	6,96	83,3
148,8	19	11,4	9,3	167	7,54	78,8
146,9	3,3	14,2	6,9	103	8,04	77,5
116	130	36,65	10,35	115	7,58	78,8
131,5	42	10,2	8,8	176	8,22	70,7
105,6	9	57,9	4,75	162	7,7	73,2
75,5	19	41,8	9,4	189	8,1	73,9
111,7	5	9,6	6,25	158	7,55	74,2
57	0,4	9,2	59,5	188	8,08	74,4
30,8	4,2	5,5	0,35	124	8,13	73,0
126,7	2,5	9,5	4,6	153	8,38	64,6
238,8	30	27,8	9,3	39	7,72	76,1
163,3	10	33,4	10,05	29	7,52	78,3
247,3	7,5	30,2	8,2	35	7,78	82,5
242,1	10,5	28,6	9,1	27	7,71	78,9
266,8	16	25,4	10,05	25	8,19	78,1
191,4	4,5	33,5	9,8	7	7,26	71,7
252,9	5,3	22	2,7	68	7,25	83,1
218,1	10	33,8	8,85	31	5,72	75,4
149,7	6,6	17,5	4,2	199	7,46	86,3
207,8	25	18,5	8,5	61	7,22	85,5
121,7	7	12,9	2,9	159	7,21	83,3
276,6	10	15,7	5,1	15	7,44	78,8
147,1	50	26,3	10,5	149	7,16	77,5
139	51	23,05	10,25	115	6,96	78,8
121,5	8,5	12,4	6,95	191	7,69	70,7
153,1	30	171	9,35	101	7,32	73,2
146	19	11,82	3	160	7,21	73,9
122,3	14	15,5	3,6	175	7,58	74,2
138,4	4,4	18,4	5,6	156	7,5	74,4
158,6	27,5	21,7	9,6	133	7,73	73,0
112,4	44	16,5	8,7	122	7,4	64,6

airtemp._°C	bodytemp._°C
8,4	37,14
10,0	37,4
11,4	37,49
10,9	37,49
10,5	37,48
5,4	36,98
9,1	36,89
10,2	37,29
9,2	37,19
9,7	37,27
9,3	37,38
8,3	37,46
9,7	37,16
7,8	37,39
11,9	37,49
14,1	37,23
12,5	37,59
12,7	37,39
12,2	37,58
8,3	37,29
12,0	37,26
8,4	37,39
10,0	37,3
11,4	37,28
10,9	36,89
10,5	37,29
5,4	37,19
9,1	37,51
10,2	37,16
9,2	36,86
9,7	37,29
9,3	37,39
8,3	36,78
9,7	37,28
7,8	37,44
11,9	36,72
14,1	37,49
12,5	37,39
12,7	37,28
12,2	37,29
8,3	37,47
12,0	37,69
	37,09

	37,08
	37,28
	37,39
	37,19
	37,29
	37,29
	37,42
	37,2
	37,19
	37,04
	37,13
	36,89
	37,14
	37,28
	36,78
	37,14
	37,29
	36,99
	37,1
	37,49
8,4	37,29
10,0	37,3
11,4	37,47
10,9	36,81
10,5	36,69
5,4	37,46
9,1	36,99
10,2	37,29
9,2	37,18
9,7	37,19
9,3	37,29
8,3	36,64
9,7	36,98
7,8	37,29
11,9	37,07
14,1	37,21
12,5	36,99
12,7	37,18
12,2	37,19
8,3	36,94
12,0	37,29
8,4	37,49
10,0	37,5
11,4	37,19
10,9	37,59
10,5	37,59
5,4	37,29
9,1	37,69
10,2	37,49
9,2	37,6

9,7	37,79
9,3	37,63
8,3	37,66
9,7	37,57
7,8	37,69
11,9	37,69
14,1	37,46
12,5	37,57
12,7	37,69
12,2	37,28
8,3	36,93
12,0	37,48
8,4	36,9
10,0	36,94
11,4	37,38
10,9	37,29
10,5	37,69
5,4	37,49
9,1	37,29
10,2	36,87
9,2	36,99
9,7	36,98
9,3	37,29
8,3	37,49
9,7	37,16
7,8	37,47
11,9	37,49
14,1	37,54
12,5	37,29
12,7	37,27
12,2	37,39
8,3	36,78
12,0	37,69
